# Respectful maternity care and mistreatment: Women’s experiences during induction of labor and childbirth in Ghana

**DOI:** 10.1371/journal.pone.0314990

**Published:** 2025-01-14

**Authors:** Kwame Adu-Bonsaffoh, Grace T. Newman, Kenneth Atobrah-Apraku, Yaw Opuni-Frimpong, Joseph D. Seffah

**Affiliations:** 1 Department of Obstetrics and Gynaecology, University of Ghana Medical School, Accra, Ghana; 2 Department of Obstetrics and Gynaecology, Korle-Bu Teaching Hospital, Accra, Ghana; Medical Research Council, SOUTH AFRICA

## Abstract

**Background:**

Most studies on respectful maternity care (RMC) and mistreatment of women have focused on intrapartum care with limited information on how women are treated during induction of labor (IOL), pre-labor phase of the maternity care continuum. Emerging multi-country evidence indicates that nearly 30% of women who undergo IOL do not consent to the procedure and constitutes a violation of their rights to optimal maternal health. This study explored women’s lived experiences of respectful care and mistreatment during IOL in a tertiary setting in Ghana.

**Methods:**

This was a qualitative phenomenological study conducted between September 2021 to October 2021 in Ghana. The eligibility criteria comprised women, aged ≥ 18 years who underwent IOL with singleton gestations. Purposive sampling was employed in recruiting the study participants (n = 17). Data analysis was performed based on thematic content using the inductive qualitative analytic framework approach.

**Results:**

Nearly all the participants (94.1%) were first-timers to IOL. In general, we determined mixed findings relating to the experiences of RMC (respectful versus disrespectful care). Some women experienced respectful care including effective communication, optimal counseling and appropriate professionalism resulting in adequate client satisfaction with care. Conversely, we determined that some mothers experienced mistreatment of different types during labor induction and birth including verbal abuse, lack of privacy, neglect, ineffective communication, inadequate pain relief, non-consented care and inadequate professional standards. There were no reports of physical abuse. Mixed responses (positive and negative) were heartily described concerning future utilization of the health facility considering the quality of care they received. Personalized recommendations to improve the quality of care during IOL were provided by the affected women and these summed up to RMC (e.g. effective communication, adequate analgesia, shared-decision making).

**Conclusion:**

Our study indicates that women experience varied forms of mistreatment during induction of labor and childbirth, and can be potentially traumatic psychologically considering their prolonged exposure to health facilities. Context specific strategies to expedite integration and adherence to RMC guidelines in maternity care are recommended to improve the quality of care during induction of labor and birth.

## Introduction

Respectful maternity care is defined by the World Health Organization (WHO) as “the organized care provided to all women in a manner that maintains their dignity, privacy and confidentiality, ensures freedom from harm and mistreatment and enables informed choice and continuous support during labor and childbirth” [1, 2]. Globally, RMC is considered a vital evidence-based strategy to improve women’s utilization of maternity care services and satisfaction with care [1–3]. Given the sensitive nature of how some women are mistreated during childbirth and the implications on future utilization of health facilities, effective provider-client communication remains the cornerstone of RMC especially in LMICs where access to health care is heavily influenced by culture, society, demography and religion among other factors [3, 4].

Globally, there is evidence that laboring women experience significant levels of mistreatment and disrespectful care and the impact can be potentially detrimental in LMIC where health inequity is more pervasive [2–4]. In a recent multi-country study on “how women are treated during childbirth” over 35% of postpartum women experienced mistreatment which was also directly observed in approximately 41% of laboring women [5]. Different typologies and severities of mistreatment have been described: physical (slapping, kicking, pinching and gagging), verbal (use of rude or harsh language, threats, blaming for poor outcomes and accusatory comments), lack of privacy and confidentiality, stigma and discrimination, neglect, ineffective communication, non-consented care and lack of professional standards [4, 6].

Recent evidence indicate that disrespectful care is more common in women who undergo more obstetric procedures or interventions [5, 7]. A typical example of life-saving obstetric interventions is induction of labor, defined as the artificial stimulation of uterine contractions prior to spontaneous or natural labor initiation with aim of achieving vaginal birth [8–10]. It is a common maternity care intervention to facilitate normal childbirth and avert cesarean section, a major surgical procedure to deliver babies. IOL is a pre-labor obstetric intervention that predates active labor and childbirth in the continuum of maternity care. It is therefore considered as the preparatory or transitory phase of active labor and requires adequate maternal education, counselling and psychological preparation for the procedure [11].

However, IOL can potentially result in significant maternal and neonatal adverse outcomes and therefore requires extremely careful monitoring for both the mother and the fetus [8, 11]. In high income countries, women who are undergoing IOL are monitored continuously to ensure early identification of complications and implementation of salvage procedures including emergency surgery to avert adverse outcomes. In LMICs, the story is quite different as the luxury of continuous fetal monitoring is not always achievable and hence high rate of complications [11]. Recent evidence indicates that induction of labor is a challenging obstetric event for childbearing women due to lack of adequate information about the induction process, inadequate informed consent and insufficient support during the induction process [12, 13].

Most studies on mistreatment of women have focused on labor and childbirth, and there is limited information on how women are treated during pre-labor phase of the maternal care continuum including IOL. Evidence from the recent WHO multi-country study on respectful maternity care indicates that about 27% of women who underwent IOL did not consent for the procedure and constitutes a major violation of their rights [5]. Given the need for continued presence and clinical monitoring (maternal and fetal) by health workers during induction of labor (prolonged interpersonal interactions) and the potential for further surgical procedures, this study explored women’s lived experiences of respectful care and mistreatment during induction of labor and childbirth at a tertiary setting in Ghana

## Methods

### Study design and site

This was a qualitative study conducted between 1^st^ September 2021 to 31^st^ October 2021 at the Department of Obstetrics and Gynecology (Maternity unit) of the Korle Bu Teaching Hospital (KBTH) in Accra, Ghana. Using phenomenology as the main qualitative approach [14] we explored women’s lived experiences of respectful maternity care from the time of induction of labor to childbirth in the tertiary hospital. The study site is the largest tertiary hospital in Ghana with about 10,000 births every year and induction of labor accounts for significant proportion of deliveries in the hospital [11]. This qualitative synthesis was part of a larger mixed methods study on induction of labor at KBTH. In this paper, we present women’s experiences of mistreatment during induction of labor through to childbirth. Further details of the methods including women’s lived experiences during labor induction have been published elsewhere [15].

### Study population

The eligibility criteria comprised women aged ≥18 years who had recently undergone induction of labor for singleton pregnancies at the Maternity of KBTH. We excluded women who were referred to KBTH after delivery even if induction of labor was carried out for them before they delivered. We also excluded women who carried multiple gestations.

### Data collection and sampling

Purposive sampling was employed in recruiting the study participants. This is a non-probability sampling involving identification and recruitment of appropriate research participants based on the objectives of the study [16]. In-depth interviews (IDIs) were used to obtain a methodical understanding of women’s experiences during the process of labor induction to childbirth with a special focus on respectful maternity care and mistreatment women. The use of IDIs generates an all-inclusive understanding of human phenomenon grounded on lived experiences and perspectives [17]. In this study, we engaged an experienced qualitative-based research assistant who conducted all the qualitative interviews with the aid of an interview guide (S1 File). The research assistant was not a health worker, not related to any of the respondents and had BSc degree. We initially conducted the interviews in English or “Twi” and all the IDIs were audio-recorded. Twi (Akan) is the most widely spoken local language in Ghana. The IDIs were conducted in private rooms at the Maternity unit of the hospital and the duration of each interview was approximately 30 to 45 minutes. The IDIs were continued alongside transcription and translation until data saturation was reached at which point no new themes emerged with further interviews.

### Ethics approval and consent to participate

Ethical approval for this research project was obtained from the Ethical and Protocol Review Committee of the College of Health Sciences University of Ghana (Protocol ID: CHS-EtM.2-P5.5/2020-2021). Also, we ensured anonymity by non-inclusion of any identifiable information on the study participants. In addition, all pregnant women included in the study provided written informed consent before the in-depth interviews were conducted.

### Data management and analysis

The research assistant and one author (KAA) translated the Twi audios into English and then transcribed all the audio interviews immediately after the IDIs. The transcripts were double-checked and validated for completeness and accuracy by the principal investigator (KAB). Data analysis was performed based on thematic content comprising identification of relevant themes or patterns within the data [18]. We employed inductive qualitative analytic framework approach (“bottom up”) in identifying the common themes that emerged from the transcripts [18]. Thus, the themes were generated via recursive reading of all the transcripts to identify the relevant themes that emerged. We ensured triangulation of the results by including pregnant women with different indications for the induction of labor such as preeclampsia, postdate and gestational diabetes (data source triangulation) [17]. The participants were also treated by different groups of health workers (obstetricians and midwives) with varied level of clinical experiences at the Maternity unit of the hospital. The consolidated criteria for reporting qualitative research (COREQ) [19] was used as a guide in producing this article.

## Results

In this study, 20 women who underwent induction of labor were invited to participate in the IDIs out of which 3 declined resulting in 17 participants who were interviewed. The main indications for induction of labor are indicated in Table 1. The socio-demographic characteristics of the study participants are indicated in Table 2: most of the participants were in the age group 25–34 years (52.9%; n = 9) and had at least secondary level education (52.9%; n = 9). Among the women who had induction of labor performed for various indications, 12 (70.6%) achieved vaginal birth whilst 5 (29.4%) were delivered via cesarean section (CS). Induction of labor was a new experience to nearly all the women (94.1%, n = 16); only one woman (5.9%) had previously experienced IOL.

**Table 1:** Indications for induction of labor and mode of birth.

Indication	Number N = 17	Percentage (%)	Mode delivery
Postdate/prolonged pregnancy	8	47	7 Vaginal 1 Cesarean
Pre-eclampsia	5	29.4	2 Vaginal 3 Cesarean
Gestational diabetes	2	11.8	2 Vaginal 0 Cesarean
Sickle cell disease	1	5.9	Vaginal
Fetal anomaly	1	5.9	Cesarean

**Table 2:** Sociodemographic characteristics of participants.

Variable	Number (n)	Percentage (%)
**Age (years)**		
<20	1	5.9
20–24	3	17.6
25–29	5	29.5
30–34	4	23.5
≥35	4	23.5
**Marital status**		
Single	2	11.8
Married/co-habiting	15	88.2
**Education**		
None/primary	2	11.8
Secondary School	9	52.9
Tertiary	6	35.3
**Number of previous births**		
Nulliparous (0)	7	41.2
Primiparous (1)	4	23.6
Multiparous (≥2)	6	35.2
**Ethnicity**		
Akan	13	76.4
Other	4	23.6

The prominent themes that emerged from interviewing the participants (women who underwent induction of labor) comprise the following:

1. Indications of labor induction
2. Experience of disrespectful care and mistreatment
3. Future utilization of health facilities
4. Women’s recommendations on induction of labor

### 1. Indications for labor induction

Nearly all the study participants were knowledgeable about their individual indications for which they underwent induction of labour. The women were adequately informed about the respective reasons for the induction of labor. Most of the women had IOL on account of postdate (47%) followed by preeclampsia (29.4%). Other important indications were sickle cell disease and gestational diabetes. All the women who had IOL due to prolonged pregnancy or postdate were able to describe the circumstances surrounding their delivery (indications for IOL)

*“The main reason was that my time was due on 10*^*th*^ *September and the doctor said he was going to give me an extra one week after which if I haven’t delivered, he was going to do the induction”* (32 years, married, Vaginal birth)

*“My time had passed so they referred me here*. *I was admitted first for them to observe me if the baby would come but nothing happened the first day so they inserted the medicine and then monitored me throughout the day*. *I delivered by 7pm*” (35 years, married, Vaginal birth)

*“I was admitted due to postdate*. *Since I was admitted there hasn’t been any bad experience*. *I came on Sunday and then the procedure was done the next day*. *They inserted a drug underneath me [intravaginally] and then they sent me to the labor ward where I was monitored for about 2 hours before I delivered”* (28 years, married, Vaginal birth)

*“I was 41 weeks and 3 days and the baby wasn’t coming*. *When I came here [hospital] they introduced induction to me and I just went through the process”* (27 years, married, Vaginal birth)

Similarly, hypertensive disorders (mainly preeclampsia) constituted the second major reason for induction of labor and the affected women had adequate knowledge about their medical conditions.

*“When I went there, they told me my [blood] pressure was high so they were transferring me to Korle Bu hospital. When I came here [Korle Bu], they checked my BP regularly for some time and told me they would admit me. I was given some injections. Around 4pm to 6pm, they gave me the medicine to insert under my tongue and they sent me to the ward”* (36 years, married, Cesarean birth)

*“He [the doctor] said they have to monitor me up to 21*^*st*^ *of this month if the BP will come down or remain high and they will do forced labor*. *That is exactly what they did*. *I was here and on the 21*^*st*^ *they did the forced labor but it was not successful so they operated on me”* (36 years, married, Cesarean birth)

Two women (11.8%) were induced due to gestational diabetes and they accurately narrated how they went through the induction process.

*“I came here on account of gestational diabetes mellitus and also I have worsening sciatic nerve pain on my left side”* (28 years, married, Vaginal birth)

*“They said I have gestational diabetes so I am not allowed to do the full 40 weeks”* (37 years, married, Vaginal birth)

One woman had sickle cell disease which constituted the main reason for the induction of labor she underwent.

*“My due date is 31*^*st*^ *but because I have sickle cell my doctor said he will induce me for me to deliver before the due date”* (36 years, married, Vaginal birth)

### Women’s experiences of labor induction process

Most of the women described their experiences of induction of labor at the tertiary hospital including the induction procedures employed. Most of the respondents mentioned the use of medications in carrying out the induction of labor. The most prominent and recuring women’s experience was the severe pain associated with induction of labor and this was mentioned and emphasized by nearly all the respondents.

*“I knew little that there is a medicine that will be put under you [vaginal insertion] for the cervix to open and that it is very painful. Before the procedure was started, they also explained to me that they would insert the medicine to help open my cervix”* (35 years, married, vaginal birth)

*“It was normal just that the induction process is painful*. *They come and check the baby’s heartbeat*, *your BP and temperature*. *They first insert their hands [vaginal examination] to see if the womb is open or not and then they insert the drug and repeat the examination after 4 hours”* (31 years, married, vaginal birth)

Some of the women provided vivid narration of the whole process of IOL including the number of doses of the medications used for the induction, the counselling processes and the precautions given.

*“I went through the normal process. The nurses were nice. The induction was done the day after I came on admission. I received two doses before I was sent to the labor ward. In less than 20 minutes the baby was out”* (36 years, married, vaginal birth)

*“They inserted a medicine under me [vaginally] and they told me that if I don’t deliver*, *he [the doctor] will have to do an operation*. *After some time*, *the baby was not out so they went to do the operation [cesarean section]*. *The drug was inserted on Tuesday and the operation was done on Wednesday”* (18 years, single, cesarean birth)

In addition, vaginal examination experiences by the mothers were a very prominent theme that emerged. Vaginal examination during induction of labor and delivery was considered painful and unbearable by majority of the mothers.

*“The forced labor [induction of labor] was difficult. It was very painful and some of the doctors were putting their hands under me [vaginal examination]”* (36 years, single, cesarean birth)

*“The inserting of hands into my vagina [vaginal examination]… It came with abdominal pain and waist pain and it was unbearable*. *I was not feeling comfortable”* (31 years, married, vaginal birth)

Some of the women feared undergoing induction of labor mainly because of potential complications of the procedure. One participant who had not experienced labor induction before indicated she was still panicking even after receiving appropriate counselling and assurance from the health workers.

*“I was scared because I have never had the forced labor before. I told one nurse and she told me it is not scary and it is essential for the delivery of the baby. I understood her but I was still panicking”* (28 years, married, vaginal birth)

### 2. Experience of disrespectful care and mistreatment

Women’s narratives concerning “how they were treated” during the induction of labor were mixed (positive and negative experiences). Some described experiences of respectful care whiles others experienced some forms of mistreatment. The areas of respectful care and mistreatment described by the participants include privacy and dignity, birth companion and emotional support, provider-client communication, verbal abuse, neglect, informed consent issues and lack of professionalism.

### Privacy and dignity

Maintenance of privacy and dignity was an important sub-theme that was considered very important to the birthing mothers. There were mixed reports concerning how the dignity and privacy of the women who were undergoing induction of labor were maintained. Some of the mothers were (dis)satisfied with the level of care they received with respect to maintenance of their dignity and privacy as indicated in the quotes below:

*“Here in the maternity ward, they treated me with respect. It is the labor ward that I was disrespected. But I feel they did not preserve my dignity because when my cloth fell off during the examination, they didn’t pick it up for me and the left me exposed. So as for my dignity I feel they did not preserve it*” (36 years, single, Cesarean birth)

*“The first floor I was sent to*, *they were not opening my nakedness to the public*. *The second floor I went to it was the same*. *The doctors had a screen they used to cover the space I was in*. *But here [main ward]*, *it is open and those around you can see everything*” (36 years, married, Cesarean birth)

On the other hand, some of the women reported that they experienced respectful maternity care optimally including utmost respect and preservation of their dignity and privacy during the labor induction and birthing processes. In addition, some participants described how they were adequately counselled and educated by the health workers prior to clinical procedures. These demonstrations of respectful care by the health workers during induction of labor and childbirth facilitates women’s satisfaction with the care they received.

“*They use your cloth to cover you when they are examining you. At the side ward, they don’t use any curtains and I think the cloth is enough to preserve our dignity”* (28 years, married, vaginal birth)

*“As for this place they really respect and treat people with dignity*. *Before they examine you*, *they tell you what they are about to do so that you can cover yourself”* (28years, married, vaginal birth)

*“The doctor in charge was very kind and nice*. *He took me through the process*. *Whenever he is examining you*, *he tries to have a chat with you to take your mind of the pain or any other thing*. *Before you realize he’s done with whatever he wants to do”* (32 years, married, vaginal birth)

Although most of the women (76.5%, n = 13) were satisfied with the care they received, some participants hinted that there were major gaps in the quality of care they received resulting in negative experiences. Some participants felt they were not treated well especially with the care they experienced at the labor ward. Lack of privacy was paramount. Several laboring women were admitted at the same room without curtains and they were made to cover themselves with cloths during vaginal examinations. Although, they used their cloths to cover themselves during vaginal examinations, the mothers lamented on the lack of curtains or partitions between the women as they lay on their beds in an open ward. They indicated that lack of curtains constituted significant limitation in the care they received. However, most of the participants were “okay” with the care they received.

“*I did not see any form of curtains partitioning the place. They only covered us and it was okay for me*” (35 years, married, vaginal birth)

“*They make us cover ourselves with cloths when examining us*. *What I did not see is the covering with curtains” (29 years*, *married*, *vaginal birth)*

“*They don’t have curtains here*. *At the labor ward they had the curtains and covered me before attending to me*. *But I think they cared for me and my baby with respect and dignity*. *I won’t say it was sufficiently done”* (31 years, married, vaginal birth)

### Birth companion and emotional support

An important sub-theme that emerged was women’s constant request for birth companions especially during labor when they were in severe pain. Birth companion of choice was one of the main support most of the respondents requested for during labor. However, it was evident that the parturient did not have birth companions and the health workers could not adequately provide the needed support partly because of the busy duties, high patient load or the design of the labor wards where multiple laboring women are cared for in the same room. The quest for birth companion during the IOL and childbirth is exemplified by the experience narrated by a teenage mother as indicated below:

*“When you even call someone to hold you, the person will not even come. There was someone [health worker] who when even she is around you and you tell them you want to hold them they say no*” (36 years, single, Cesarean birth)

Relatedly, the need for emotional support was considered paramount to some of the women who underwent induction of labor. However, most women narrated that their relatives were not allowed to provide the needed emotional and psychological support. One woman indicated that her husband was her main source of emotional support, but she was denied the opportunity of receiving the needed companionship from her husband. Another participant indicated she did not have any emotional support and any complaints she made was responded to as normal.

“*Normally when the relatives come to visit us, sometimes the nurses will come and sack them. Almost every day when my mum comes, she has a problem with it. When she comes, I prefer she stays for a little bit longer before she goes back. Whenever the nurses come in, they also want them [relatives] to leave, and she feels bad that she has to leave me. And she is also taking care of my first child at the same time*” (28 years, married, Vaginal birth)

“*My husband has been my main source of emotional support*. *I did not want to talk to someone who will say something hurtful to me because I looked up to them*” *(36 years*, *married*, *vaginal birth)*

“*There was no emotional support*. *Any complaint I made they will tell me it’s normal”* (31 years, married, vaginal birth)

### Pain relief during childbirth

Some of the respondents indicated the need for adequate pain relief during the labor induction process and labor. There was evidence that most laboring women experienced severe pain that was generally considered as normal by the health workers who encouraged them to tolerate the labor pain. Some participants recommended the need for adequate pain relief for laboring women to reduce severe pain they experience. These reports are indicative of inadequate analgesia for women during labor induction and childbirth, requiring urgent attention

“*They [health workers] should get painkillers. Because yesterday the pain was too much and I asked for painkillers and the nurse said we don’t take painkillers here because the pain is normal. So, if they can get any medicine that will reduce or numb the pain for us that will be a good way to go” (32 years*, married, vaginal birth)

*“Even though the insertion of the medication is uncomfortable*, *there is nothing wrong with it*. *I was even asking for pain relief and he [the doctor] told me if he injects me all will be ok with me but the baby will not come out well”* (27 years, married, vaginal birth)

Not surprisingly, other women were aware of epidural analgesia use in labor wondered why it is usually not provided to women in labor. This observation by the women and their awareness of the relevance of epidural analgesia is call for health workers to provide adequate pain relief during childbirth.

“*I learnt there is epidural that can lessen the pain but nothing like that was given here” (*26 years, married, Vaginal birth*)*

### Provider-client communication

An important finding recounted by the women was lack of adequate communication between the women and health workers. One woman lamented that a lot of doctors entered her room without any formal introduction and she wondered their identity and where they were coming from. There were instances where women were enrolled into a study without proper informed consent.

“*So many doctors have entered the room [ward] unaccompanied and we don’t know who or where they came from. Can you imagine one person came that he is doing a study and he wanted me to urinate for him to a test? One too came and took my folder. My problem is they should let us know what they will do for us when we come on admission*” (28 years, married, Vaginal birth)

In addition, one mother recounted her negative experiences with the providers as they failed to ask her questions related to her socio-economic background. She added that such interrogation or inquiry on patients’ social and economic background could proffer health workers adequate information on women’s socio-economic capabilities in terms of their ability to afford their health care cost.

*“They communicated with me, but they never asked about personal issues like what work my husband and I do and whether we will be able to take care of my hospital bill. I don’t think it was effective”* (36 years, married, cesarean birth)

There is evidence that pregnant women experience significant financial challenge which markedly influence the care they receive. For instance, the same mother described her experience of being detained at the hospital following her discharge from the hospital due to her family’s inability to pay for cost of her hospitalization

*“It was not easy. All my money got finished. We were discharged but we have not been able to clear our bills. I called my husband and we are still looking for some help to clear the bills. My stay here used up all the money we had. I used to be a trader and my husband is a janitor at Fidelity bank. He has taken so many loans since I came on admission. We have no one to help us.”* (36 years, married, Cesarean birth)

On the other hand, some women experienced perfect provider-client communication including adequate counselling and education

*“Prior to the procedure [induction of labor] I did not know anything about it but they explained everything in detail to me before it was done”* (36 years, married, Cesarean birth)

“*The care so far has been excellent starting from Saturday when I came on admission*. *The nurses received me very well and even took me round to show me the washroom and where they wash the bowls and other things”* (32 years, married, Vaginal birth)

### Verbal abuse

Nearly all the women indicated that they received respectful care in terms of verbal abuse. However, few women mentioned some occurrences of verbal abuse during the induction and the birthing processes. For example, one woman described an experience where the nurse who transferred her to the labor ward spoke to her in a “high tone” voice. However, she attributed the nurse’s behavior to the excessive noise she (the woman) was making due to the severe pain she was experiencing following the induction of labor. She attributed the severe pain to the medication used for the IOL.

*“When I was in labor the nurse who was transferring me to the labor ward was speaking in a high tone. I suppose it was also making a lot of noise because of my pain. I wanted them to send me quickly because I was in pain due to the drug that was inserted [intravaginally] for me and they were delaying”* (21 years, single, vaginal birth)

In addition, other women witnessed how some teenage mothers were verbally abused on the ward. This observation indicates that teenage mothers experience high occurrence of verbal abuse.

*“I want them to get very close to us and ask about our support system and how we are taking care of ourselves. I observed how teenage mothers were spoken to harshly because they were not getting the needed support to take care of themselves here. They should rather be helping if they have any help. The doctors and nurses should not be arrogant because of their education and only relate well with educated people. The teenage mothers also deserve respect”* (36 years, married, cesarean birth)

### Neglect

Some of mothers who underwent induction of labor narrated their varied experiences concerning whether they were neglected or not during the induction process. Whiles some parturient experienced an excellent care from the health workers others reported negative experiences of how they were neglected during the process of induction and childbirth.

*“When I was sent to the labor ward, I saw that the doctor who was taking care of me on the ward was already talking to a doctor in the labor ward and they told me to hold on for a moment. I did not feel neglected because my ward doctor was already there talking to the labor ward staff to give me a bed”* (21 years, single, vaginal birth)

*“I was just screaming and they were telling me I should calm down*. *Some of the midwives are good and some are not good*. *When you call someone to help you the person will never come*. *They will tell you that bear the pain*. *I will say the care was good*, *somehow”* (36 years, married, cesarean birth)

“Nobody abused me physically. I was left alone at a point and I was very angry. I felt they should give me attention but they could not” (36 years, single, cesarean birth)

*“I think the time wasn’t exactly up that is why they were not minding me”* (28 years, married, vaginal birth)

### Consent for induction of labor

There were mixed report concerning obtaining informed consent for induction of labor and other related procedures. Some women indicated that they received the needed information on induction of labor prior to consenting and signing the informed consent form. Thus, there was evidence that some of the mothers had adequate understanding of the induction process and opted for it.

*“The doctor told me that for forced labor, it is very painful and that my womb has to open about 9 times but mine couldn’t open up to that 9. I was at my 4*^*th*^ *when they said it was not working so they have to take me to theatre”* (36 years, single, Cesarean birth)

*“The procedure was also explained to me for me to understand it well and I signed the consent”* (28 years, married, Vaginal birth)

On the other hand, few women indicated they did not receive the needed information although they signed for the procedure

*I didn’t know anything until the nurse that transferred me here told me they will do induction*. *Before the process started*, *they did not explain the details for me*. *They just said they will insert the medicine [intravaginally]*. *They made me sign a form”* (31 years, married, Vaginal birth)

### Lack of professionalism

Some of the participants felt the level of professionalism was suboptimal for some of the health workers. One participant observed that some of the providers do not adequately triage cases at the point of admission and end up not attending to emergency cases promptly. She recommended that providers should attend to urgent cases needing immediate attention before attending to the relatively stable clients.

*“I think they should prioritize their work and handle more urgent cases before more the stable ones”* (37 years, married, Vaginal birth)

In addition, another mother recounted how she was wrongly tagged for cesarean section when she had actually been admitted for induction of labor. She was mistaken for another patient who had been admitted for cesarean section. They started preparing her for cesarean section until she hinted the providers that her reason for admission was induction of labor.

*“Three people [health workers] came to tell me I am doing CS, not knowing it is not my name. It was Mercy instead of Esther and they were all here packing my things for CS until I told them I am Esther not Mercy and they told me they were sorry. Then they should check the name on the system to know what they are about because you can’t come and tell me I am doing CS when I am here for induction of labor. The nurses have to be trained seriously”* (28 years, married, Vaginal birth)

### 3. Future utilization of health facilities

There were mixed responses concerning their utilization of the health facility in future considering the care they experienced during their current hospitalization. Majority of the respondents preferred to receive care in the same facility when they get pregnant again. This is a likely indication they received optimal maternity care. The reasons cited by the women include availability of equipment especially in times of emergencies and doctors compared to other hospitals.

“*I will still prefer this same facility. Actually, I don’t normally like the private hospitals because most times they don’t have many doctors around and you know that with childbirth anything can happen. We don’t pray for complications though but that is my mentality*” (32 years, married, Vaginal birth)

*“I will come here again*, *only I am done having my children*. *But for a sister of mine asking for advice*, *I will have them come here because even at the private facilities they refer here when there are complications so why don’t you come here directly*. *The way they take care of us with love makes you*, *the patient*, *feel good and not think about the issue for which you were brought here”* (36 years, married, Cesarean birth)

However, some of the participants (23.5%, n = 4) indicated total dissatisfaction and their determination not to seek maternal care at the hospital due to their perceived substandard care. Reasons provided for seeking care elsewhere related to inappropriate health workers’ behavior and suboptimal care. One mother expanded on the reason why the care in government hospitals maybe suboptimal: lack of appropriate punishment for health workers’ poor attitudes and inappropriate behavior or professionalism.

*“I will go somewhere else. I think when you go to a private hospital, the care there is better than a government hospital. Over there if you are a nurse and you misbehave and you are reported you will lose your job but over here in the government sector, even if someone is reported the matron will say one or two things; nothing happens and at the end of the month their salaries come. So, it is like everybody is doing what he or she likes but at the private sector because they know they can lose the job they tend to work harder”* (28 years, married, Vaginal birth)

Three (17%) of the respondents stated openly they will not want to deliver in the facility again. One mother cited her experience of maltreatment at the labor ward as her reason. In addition, one participant narrated how she was ignored and disrespected when she needed help during labor. On the other hand, one mother decided not to access maternity care at the facility because she lived far from the hospital and transportation is expensive

“*I am not driven to come here again because of the way they maltreated me at the labor ward. The nurses disrespected me”* (36 years, single, Cesarean birth)

“*I don’t want to come here because of the distance*. *I only came because they said the issue with my baby could be handled here*. *But I would not want to be here again because of the distance and the transport fare–it is expensive*.*”* (18 years, single, Vaginal birth)

### 4. Women’s recommendations on induction of labor

The participants provided some context related recommendations based on their lived experiences during induction of labor and delivery. One of the main themes concerning recommendations relates to measures to improve respectful care by the health providers.

*“You have to talk to your nurses to respect–those who are up and coming and under training”* (36 years, single, Cesarean birth)

*“I will not recommend induction to any woman*. *The drug is not the issue but the insertion of the fingers of the doctors [vaginal examination]”* (31 years, married, Vaginal birth)

One mother recounted her experience of good communication with the providers including how she was assured constantly that she would deliver her baby successfully. However, she felt the providers did not factor her suggestions or contribution into the decision making. She therefore felt suboptimal provider-client communication. This exemplifies the importance of shared decision making to improve women’s intrapartum experiences.

*“Communication between me and the doctors was effective. They told me they don’t just get up and do CS [cesarean section] but it did not answer my needs then. I liked how they constantly assured me that I can deliver. But when I felt at a point that I couldn’t continue, I think they should have listened to me*” (31 years, married, Vaginal birth)

To buttress the recommendation for shared decision making, one mother openly recommended that health workers should actively consider the opinions of patients before carrying out procedures on them. This recommendation underscores the relevance of shared decision making to the patients as it makes them feel active participants of their own treatment

*“I will suggest that when the doctors are taking decisions, they should take the opinion of the patients and explain the whole procedure [induction of labour] to the person before starting it”* (28 years, married, Vaginal birth)

Provision of adequate pain relief was an important subtheme that emerged repeatedly. Majority of the participants implored maternal health workers to provide pain relief for women during labor induction and childbirth processes. One mother speculated if the severe pain women experience during labor was a form of punishment meted to them by health workers. This assumption was based on health workers’ reluctance to provide adequate labor analgesia for women even when they request for pain relief.

*“My main recommendation is pain relief. When you ask for a pain relief they don’t give and it is as though I was under a punishment”* (37 years, married, vaginal birth)

One clinically relevant recommendation was the need for the health workers to spend adequate time with the women to optimally explain the procedures and processes (including medications) for them to appreciate the care they receive. For instance, one mother recommended that the doctors should devote some time to explain how the medications they prescribe work and their possible side effects.

*“I know the doctors know best but I think they should spend time explaining the drugs they will give us and its effects to us”* (35 years, married, vaginal birth)

## Discussion

In this qualitative study, we explored recently delivered mothers’ lived experiences of (dis)respectful care and mistreatment during induction of labor and childbirth in Ghana. Women reported mixed (respectful versus disrespectful) experiences of care. We determined that women experienced mistreatment of different types during the process of labor induction and birth: verbal abuse, lack of privacy, neglect, ineffective communication, inadequate pain relief, non-consented care and inadequate professionalism. There were no reports of physical abuse. Women provided personalized recommendations to improve the quality of care during induction of labor and this summed up to respectful maternity care (e.g. effective communication, adequate analgesia, shared-decision making). In addition, majority of women experienced respectful care which is commendable.

Disrespectful or abusive treatment during childbirth is an indicator of poor maternity care with direct negative impact on future health seeking behavior. Current evidence indicates that women experience varied types of mistreatments during childbirth in health facilities [4, 5, 20]. In previous studies, as high as 98% of women reported disrespectful or abusive care during maternity care in Nigeria [21] and 20% in Kenya [22]. In the current study, most of the women were satisfied with the care they received and there were no reports of physical abuse. However, some mothers (23.5%) expressed dissatisfaction with the care they received and cited lack of privacy especially when multiple laboring women were admitted at the same labor ward with no curtains or partitions. The issue of childbirth-related lack of privacy and confidentiality in LMICs needs to be revisited to improve women’s experiences and satisfaction with care.

In contemporary maternity care, birth companions for laboring women are considered evidence-based intervention to improve birth outcomes when permitted in labor rooms [23]. In this study, there was evidence of considerable requests by laboring women for birth companions especially when they experienced unbearable labor pain, however, none of the women was provided with birth companions of their choice. This was partly due to the restricted labor rooms which are not large enough to accommodate the laboring woman, the midwife and birth companions. Also, the labor wards are usually occupied by multiple laboring women and the relatives or companions of all the parturient cannot be allowed into the labor ward. Therefore, restricting access to birth companions is mainly to protect and preserve the confidentiality, privacy and dignity of the other laboring women. Relatedly, emotional support and communication have been linked with positive birth outcomes and experiences [24]. Lack of emotional support, inadequate analgesia and poor communication were some of the reasons why some respondents in this study thought they were not fully satisfied with the care they received.

The quality of care people receive determines whether they will later seek care in the facility again or not. Considering the care they received during their current hospitalization, there were conflicting reports regarding whether the women would seek medical attention again in the facility. Most women still opted to have their future antenatal care at the facility because of the availability of equipment and large numbers of doctors at the tertiary center. Prior to their current hospitalization, some respondents had made decisions not to seek care in the hospital because they believed the care would be subpar due to poor attitudes of the medical staff based on other women’s experiences. However, some women indicated their strong disinterest in receiving care at the hospital due to their experiences of mistreatment and disrespectful care during the process of labor induction and childbirth. Also, long distance between their residence and the location of the hospital constituted a barrier to accessing care at the hospital.

Relatedly, there was frequent requests by the women for their active involvement in the decision making concerning their treatment plans. Some of the participants indicated they were not actively involved the decision-making process. The finding of inadequate involvement of women in their own personalized clinical care underscores the critical relevance of shared-decision making (SDM) in achieving positive pregnancy and childbirth experiences for women. In the process of SDM, health workers and their clients work together closely to achieve person‐centred care based on a common goal [25]. In maternity care setting, SDM improves women’s experience, satisfaction and ownership. It must be emphasized that SDM is not the same as informed consent [25]. Maternity health workers should always ensure women are actively involved in the joint decision making in all aspects of maternal care especially in obstetric procedures or interventions such as induction of labor and mode of delivery choice (vaginal birth versus cesarean section). We believe that effective provider-client communication including SDM is a key to achieving RMC and optimal quality of maternal care. Thus, SDM can potentially minimize mistreatment and enhance women’s satisfaction thereby incentivizing them to seek maternity care services.

### Clinical and research implications

Our study indicates that women undergoing induction of labor, a pre-labor phase of continuum of care, experience significant burden of disrespectful and abusive care. This finding is alarming as the next level of care (active labor/childbirth) is usually associated with significant mistreatment of women globally [4, 26, 27]. When added to the intrapartum care, the period of labor induction extends the overall birthing process and hence more interaction with health workers and health facilities. Occurrence of mistreatment during labor induction and then the actual intrapartum care can potentially make these cohort of women extremely exhausted emotionally and psychologically resulting negative intrapartum experience. In addition, there is evidence that IOL alone can potentially lead significant adverse perinatal outcome [8, 11, 28]. Mixed responses (positive and negative) were heartily described with respect to future utilization of the health facility considering the quality of care they received. The key concern of disrespectful care and mistreatment centers on the reluctance of women and their families in seeking facility-based maternity services. Delay in seeking care usually leads to real-time risk of preventable severe maternal morbidities or mortalities. Therefore, health workers and hospital managers should constantly be mindful of the double burden (stressful induction process with potential for adverse events and frequent occurrence of mistreatment during birth) and devise evidence-based interventions to improve women’s lived experiences of labor induction. We recommend that women slated for IOL be adequately prepared psychologically and emotionally for the potentially prolonged phase of childbirth with its associated increased risk of mistreatment.

Further research is recommended to determine the facilitators of mistreatment of women associated with induction of labor and context-relevant interventions to ameliorate this clinical challenge. These include appropriate integration of emotional and psychological support including allowing women access to birth companions of choice during induction of labor and childbirth. Multiple vaginal examinations during induction of labor with further assessments during active labor can be potentially traumatic physically and psychologically [26, 29, 30]. Health workers, therefore, require the requisite skills and (re)training to be more empathic and provide equity-but not equality-oriented care for women undergoing IOL. In addition, interventions to reduce the frequency of digital vaginal examinations such as the use of transperineal ultrasound can be explored for their applicability in contemporary maternity care practice. There is evidence that transperineal ultrasound assessment of progress of labor is more convenient, less painful and more acceptable compared to digital vaginal examination [31, 32]. This emerging intervention should be further explored for their applicability and subsequent integration in contemporary obstetric care to minimize digital vaginal examination-related mistreatment that women experience.

### Strengths and limitations

The main strength of this study relates to the assessment of respectful maternity care during the pre-labor phase of the continuum of maternity care. Most previous studies of RMC are limited to the labor or childbirth and have not fully considered the transition between antenatal and intrapartum phases of the continuum of care. In addition, the use of IDIs provided significant insights into the lived experiences of disrespectful care by women who underwent IOL. Also, we included women with varied obstetric characteristics (nulliparous versus multiparous) and different clinical indications (case mix) for labor induction (postdate, hypertension in pregnancy, diabetes). The comprehensive case mix provides a wider spectrum of data source and women’s experiences.

The main limitation of the study centers on the use of a single interviewer for all the IDIs without a note taker. The use of single interviewer might have limited the scope of probing and with increased potential of interviewer bias. However, the interviewer had the relevant experience in qualitative interview and hence less likelihood for a bias. In addition, the use of single study site can potentially limit the applicability of the results nationally as different levels of health facilities present different health system challenges with varied women’s experiences.

## Conclusion

Our study has highlighted women’s lived experiences of varied forms of mistreatment during induction of labor and childbirth in a tertiary hospital in a LMIC. These include verbal abuse, lack of privacy, neglect, inadequate pain relief, ineffective communication, non-consented care and inadequate professional standards. Women’s experiences of mistreatment during induction of labor (pre-labor phase) and subsequently in the active labor phase is considered a double burden and can be potentially traumatic psychologically resulting in negative intrapartum experience. There is the need to facilitate adequate client education and psychological preparedness towards labor induction and birth. Women described mixed responses (positive and negative) concerning their future utilization of the health facility considering the care they experienced. Strategies to expedite improvement in health system structures and policies to facilitate positive pregnancy and childbirth experiences for women are strongly recommended. Further research is recommended to explore how best to implement and maintain evidence-based guidelines on RMC into routine care for women undergoing induction of labor and birth.

## Supporting information

S1 File Interview guide. (PDF)

S2 File Supporting data. (PDF)

## Abbreviations

COREQ: Consolidated Criteria for reporting qualitative research
CS: Cesarean Section
IDI: In-Depth Interview
IOL: Induction of Labor
KBTH: Korle Bu Teaching Hospital
LMICs: Low- and Middle-Income Countries
RMC: Respectful Maternity Care
SDM: Shared-Decision Making
WHO: World Health Organization
