# Supplementary material for: Respectful maternity care and mistreatment: Women’s experiences during induction of labor and childbirth in Ghana
Source: PLoS One. 2025 Jan 14;20(1):e0314990. doi: 10.1371/journal.pone.0314990 (PMC11731704; doi:10.1371/journal.pone.0314990)
Supplement: S1 File — (PDF) [file pone.0314990.s001.pdf]

Interview guide for women who underwent induction of labour

- Could you describe why you were admitted to the hospital?
- Could you describe how you were cared for during your admission at the hospital?
- How did you experience the care you received at the hospital?
- How did your admission at the hospital affect you and your family?
- During your admission you've interacted with others (Probe further: medical professionals, your family, other patients) how do you relate to them? Did any conflicts occur, why?
- What is the most difficult aspect of the care you received at the hospital?
- In your opinion, did you experience any disrespectful care (Probe further: verbal abuse, physical abuse, discrimination, neglect, professionalism etc)?
- Prior to the procedure, did you know about induction of labour?
- Do you think there were adequate number of doctors, nurses and other professionals available to take care of you and other patients? (Probe further)
- In your opinion, did you receive the care you expected to receive at the hospital? (Probe further)
- In future, would you prefer to be cared for again in the hospital or you would prefer elsewhere if you had the opportunity? (Probe further)
- Do you think you were checked or monitored regularly as expected during the process of induction? (Probe further)
- Do you think you were able to do all the laboratory tests requested for you? (Probe further)
- What recommendations do you suggest to help improve the quality of care for women undergoing induction of labour at the hospital?
