# Supplementary material for: Respectful maternity care and mistreatment: Women’s experiences during induction of labor and childbirth in Ghana
Source: PLoS One. 2025 Jan 14;20(1):e0314990. doi: 10.1371/journal.pone.0314990 (PMC11731704; doi:10.1371/journal.pone.0314990)
Supplement: S2 File — (PDF) [file pone.0314990.s002.pdf]

Supplementary Table 1: Themes and subthemes

Subtheme	**Theme 1**<br>**Informed consent and indications for labor induction**
Postdate pregnancy	*"The main reason was that my time was due on 10th September and the doctor said he was going to give me an extra one week after which if I haven't delivered, he was going to do the induction"* (32 years, married, Vaginal birth)<br><br>*"My time had passed so they referred me here. I was admitted first for them to observe me if the baby would come but nothing happened the first day so they inserted the medicine and then monitored me throughout the day. I delivered by 7pm*" (35 years, married, Vaginal birth)
Hypertension in pregnancy	*"When I went there, they told me my [blood] pressure was high so they were transferring me to Korle Bu hospital. When I came here[Korle Bu], they checked my BP regularly for some time and told me they would admit me. I was given some injections. Around 4pm to 6pm, they gave me the medicine to insert under my tongue and they sent me to the ward"* (36 years, married, Cesarean birth)<br>*"He [the doctor] said they have to monitor me up to 21st of this month if the BP will come down or remain high and they will do forced labor. That is exactly what they did. I was here and on the 21st they did the forced labor but it was not successful so they operated on me"* (36 years, married, Cesarean birth)
Diabetes in pregnancy	*"I came here on account of gestational diabetes mellitus and also I have worsening sciatic nerve pain on my left side"* (28 years, married, Vaginal birth)<br>*"They said I have gestational diabetes so I am not allowed to do the full 40 weeks"* (37 years, married, Vaginal birth)
Sickle disease in pregnancy	*"My due date is 31st but because I have sickle cell my doctor said he will induce me for me to deliver before the due date"* (36 years, married, Vaginal birth)
Pain associated with Induction	*"I knew little that there is a medicine that will be put under you [vaginal insertion] for the cervix to open and that it is very painful. Before the procedure was started, they also explained to me that they would insert the medicine to help open my cervix"* (35 years, married, vaginal birth)<br>*"It was normal just that the induction process is painful. They come and check the baby's heartbeat, your BP and temperature. They first insert their hands [vaginal examination] to see if the womb is open or not and then they insert the drug and repeat*

	the examination after 4 hours" (31 years, married, vaginal birth)
**Vaginal examination experiences**	*"The forced labor [induction of labor] was difficult. It was very painful and some of the doctors were putting their hands under me [vaginal examination]"* (36 years, single, cesarean birth)<br>*"The inserting of hands into my vagina [vaginal examination]… It came with abdominal pain and waist pain and it was unbearable. I was not feeling comfortable"* (31 years, married, vaginal birth)
**Fear of Induction**	*"I was scared because I have never had the forced labor before. I told one nurse and she told me it is not scary and it is essential for the delivery of the baby. I understood her but I was still panicking"* (28 years, married, vaginal birth)
	**Theme 2**<br>**Disrespectful care and mistreatment**
**Privacy and dignity**	*"Here in the maternity ward, they treated me with respect. It is the labor ward that I was disrespected. But I feel they did not preserve my dignity because when my cloth fell off during the examination, they didn't pick it up for me and the left me exposed. So as for my dignity I feel they did not preserve it*" (36 years, single, Cesarean birth)<br>*"The first floor I was sent to, they were not opening my nakedness to the public. The second floor I went to it was the same. The doctors had a screen they used to cover the space I was in. But here [main ward], it is open and those around you can see everything*" (36 years, married, Cesarean birth)
**Lack of curtains**	"*I did not see any form of curtains partitioning the place. They only covered us and it was okay for me*" *(35 years, married, vaginal birth)*<br>"*They make us cover ourselves with cloths when examining us. What I did not see is the covering with curtains" (29 years, married, vaginal birth)*<br>"*They don't have curtains here. At the labor ward they had the curtains and covered me before attending to me. But I think they cared for me and my baby with respect and dignity. I won't say it was sufficiently done" (31 years, married, vaginal birth)*
**Birth companion and emotional support**	"*Normally when the relatives come to visit us, sometimes the nurses will come and sack them. Almost every day when my mum comes, she has a problem with it. When she comes, I prefer she stays for a little bit longer before she goes back. Whenever the nurses come in, they also want them [relatives] to leave, and she feels bad that she has to leave me. And she is also taking care of my first child at the same time*" *(28 years, married, Vaginal birth)*

	"*My husband has been my main source of emotional support. I did not want to talk to someone who will say something hurtful to me because I looked up to them*" *(36 years, married, vaginal birth)*<br>"*There was no emotional support. Any complaint I made they will tell me it's normal" (31 years, married, vaginal birth*
**Pain relief during childbirth**	"*They [health workers] should get painkillers. Because yesterday the pain was too much and I asked for painkillers and the nurse said we don't take painkillers here because the pain is normal. So, if they can get any medicine that will reduce or numb the pain for us that will be a good way to go" (32 years,* married, vaginal birth)<br>*"Even though the insertion of the medication is uncomfortable, there is nothing wrong with it. I was even asking for pain relief and he [the doctor] told me if he injects me all will be ok with me but the baby will not come out"* (27 years, married, vaginal birth)
**Provider-client communication**	"*So many doctors have entered the room [ward] unaccompanied and we don't know who or where they came from. Can you imagine one person came that he is doing a study and he wanted me to urinate for him? One too comes and takes my folder. My problem is they should let us know what they will do for us when we come on admission*" *(28 years, married, Vaginal birth*<br><br>*"They communicated with me but they never asked about personal issues like what work my husband and I do and whether we will be able to take care of my hospital bill. I don't think it was effective"* (36 years, married, cesarean birth)
**Effective communication**	*"Prior to the procedure I did not know anything about it [induction of labor] but they explained everything in details to me before it was done"* (36 years, married, Cesarean birth)<br>"*The care so far has been excellent starting from Saturday when I came on admission. The nurses received me very well and even took me round to show me the washroom and where they wash the bowls and other things" (32 years, married, Vaginal birth)*
**Verbal abuse:**	*"When I was in labor the nurse who was transferring me to the labor ward was speaking in a high tone. I suppose it was also making a lot of noise because of my pain. I wanted them to send me quickly because I was in pain due to the drug that was inserted [intravaginally] for me and they were delaying"* (21 years, single, vaginal birth)
**Neglect:**	*"When I was sent to the labor ward, I saw that the doctor who was taking care of me on the ward was already talking to a doctor in the labor ward and they told me to hold on for a*

	moment. I did not feel neglected because my ward doctor was already there talking to the labor ward staff to give me a bed" (21 years, single, vaginal birth)<br>*"I was just screaming and they were telling me I should calm down. Some of the midwives are good and some are not good. When you call someone to help you the person will never come. They will tell you that bear the pain. I will say the care was good, somehow"* (36 years, married, cesarean birth)
**Consent for induction of labor:**	*"The doctor told me that for forced labor, it is very painful and that my womb has to open about 9 times but mine couldn't open up to that 9. I was at my 4th when they said it was not working so they have to take me to theatre"* (36 years, single, Cesarean birth)<br>*"The procedure was also explained to me for me to understand it well and I signed the consent"* (28 years, married, Vaginal birth)
**Lack of professionalism:**	*"I think they should prioritize their work and handle more urgent cases before more the stable ones"* (37 years, married, Vaginal birth)<br><br>*"Three people [health workers] came to tell me I am doing CS, not knowing it is not my name. It was Mercy instead of Esther and they were all here packing my things for CS until I told them I am Esther not Mercy and they told me they are sorry. Then they should check the name on the system to know what they are about because you can't come and tell me I am doing CS when I am here for induction of labor. The nurses have to be trained seriously"* (28 years, married, Vaginal birth)
	**Theme 3**<br>**Future utilization of health facilities**
**Lack of personnel**	"*I will still prefer this same facility. Actually, I don't normally like the private hospitals because most times they don't have many doctors around and you know that with childbirth anything can happen. We don't pray for complications though but that is my mentality*" (32 years, married, Vaginal birth)<br><br>*"I will come here again, only I am done having my children. But for a sister of mine asking for advice, I will have them come here because even at the private facilities they refer here when there are complications so why don't you come here directly. The way they take care of us with love makes you, the patient, feel good and not think about the issue for which you were brought here"* (36 years, married, Cesarean birth)
**Poor attitudes**	*"I will go somewhere else. I think when you go to a private hospital, the care there is better than a government hospital.*

	Over there if you are a nurse and you misbehave and you are reported you will lose your job but over here in the government sector, even if someone is reported the matron will say one or two things; nothing happens and at the end of the month their salary comes. So, it is like everybody is doing what he or she likes but at the private sector because they know they can lose the job they tend to work harder" (28 years, married, Vaginal birth)
**High cost**	"*I am not driven to come here again because of the way they maltreated me at the labor ward. The nurses disrespected me"* (36 years, single, Cesarean birth)<br>"*I don't want to come here because of the distance. I only came because they said the issue with my baby could be handled here. But I would not want to be here again because of the distance and the transport fare – it is expensive."* (18 years, single, Vaginal birth)
	**Theme 4**<br>**Recommendations on induction of labor**
**Improving respectful care**	*"You have to talk to your nurses to respect – those who are up and coming and under training"* (36 years, single, Cesarean birth)<br><br>*"I will not recommend induction to any woman. The drug is not the issue but the insertion of the fingers of the doctors [vaginal examination]"* (31 years, married, Vaginal birth)
**Effective communication**	*"Communication between me and the doctors was effective. They told me they don't just get up and do CS [cesarean section] but it did not answer my needs then. I liked how they constantly assured me that I can deliver. But when I felt at a point that I couldn't continue, I think they should have listened to me*" (31 years, married, Vaginal birth)
**Providing pain relief**	*"My main recommendation is pain relief. When you ask for a pain relief they don't give and it is as though I was under a punishment"* (37 years, married, vaginal birth)
